# “It is not possible to go inside and have a discussion”: how fear of stigma affects delivery of community-based support for children’s HIV care

**DOI:** 10.1080/09540121.2018.1445826

**Published:** 2018-02-28

**Authors:** Joanna Busza, Victoria Simms, Chido Dziva Chikwari, Ethel Dauya, Tsitsi Bandason, Memory Makamba, Grace McHugh, Rashida Abbas Ferrand

**Affiliations:** a Department of Population Health, London School of Hygiene & Tropical Medicine, London, UK; b Department of Infectious Disease Epidemiology, London School of Hygiene & Tropical Medicine, London, UK; c Biomedical Research and Training Institute, Harare, Zimbabwe; d Department of Clinical Research, London School of Hygiene & Tropical Medicine, London, UK; e Independent consultant

**Keywords:** Stigma, Zimbabwe, community health worker, children, caregivers

## Abstract

Caregivers mediate children’s access to HIV care and their adherence to treatment. Support for caregivers may improve health outcomes in children, but fear of HIV stigma and discrimination can affect both uptake and delivery of support services. Within a trial evaluating community-based support for caregivers of newly HIV diagnosed children in Harare, Zimbabwe, we conducted a longitudinal qualitative study to explore how stigma affected delivery and acceptance of the intervention. We conducted semi-structured interviews with 36 caregivers, 15 children, and 20 community health workers (CHWs). Children and caregivers described experiencing or witnessing stigma and discrimination, causing some to resist home visits by CHWs. Anxiety around stigma made it difficult for CHWs to promote key messages. In response, CHWs adapted the intervention by meeting caregivers outside the home, pretending to be friends or relatives, and proactively counteracting stigmatising beliefs. As members of local communities, some CHWs shared concerns about discrimination. HIV stigma can hinder “getting a foot over the threshold” in community-based programmes, particularly for households most affected by discrimination and thus least likely to engage with services. For community support programmes to be effective, stigma-related resistance should be addressed from the outset, including CHWs’ own concerns regarding HIV stigma.

## Introduction

The negative effects of HIV-related stigma on people’s willingness to test, initiate treatment, and maintain adherence are well-documented (Katz et al., 2013; Merten et al., 2010; Parker & Aggleton, 2003). Targeted stigma reduction programmes and increasing familiarity with HIV have somewhat mitigated these effects (Stangl, Lloyd, Brady, Holland, & Baral, 2013). Widespread availability of antiretroviral therapy (ART) has further shifted perceptions of HIV from a fatal disease to a chronic condition (Chan, Tsai, & Siedner, 2015; Roura et al., 2009). Nonetheless, stigma continues to inhibit timely and appropriate engagement at every step of the HIV care cascade (Kelly, Weiser, & Tsai, 2016; Nyika et al., 2016; Treves-Kagan et al., 2016).

Children’s access to care is mediated by parents and other caregivers. Their uptake of HIV testing and ART, attendance at clinical appointments, and adherence depend on caregivers’ willingness to engage with HIV services, which can be influenced by stigma. Fear that children will be stigmatised reduces caregivers’ disclosure (Krauss, Letteney, & Okoro, 2016), and children’s ignorance of their status is associated with poorer treatment adherence (Machine et al., 2016; Nabukeera-Barungi et al., 2015). The use of community-based support to promote engagement with HIV care has been steadily increasing, often delivered by local community health workers (CHWs) (Hall et al., 2016; Jaffar et al., 2009). These programmes can reduce perceived stigma among recipients, including children (Sherr et al., 2016).

There are few accounts, however, of how stigma might affect *delivery* of community-based support, including initial acceptance by HIV-affected households. Families that might benefit from support to increase engagement may be those most likely to avoid programmes, drop out, or struggle to adopt key messages. We investigated how stigma affected a community-based intervention to support caregivers of children newly diagnosed with HIV in Harare, Zimbabwe. Specifically, we assessed children’s, caregivers’, and CHWs’ perceptions of how HIV-related stigma affected implementation.

## Study background

HIV prevalence in Zimbabwe remains high at 13.8% of the adult population, and 2.7% among children aged 10–14 (ZIMSTAT & ICT International, 2016), among whom over one third are undiagnosed (Simms et al., 2017). Children and adolescents with HIV exhibit lower engagement with health services and higher loss to follow-up (Kranzer et al., 2017). Due to the high HIV burden, local organisations increasingly rely on home based workers to deliver health care (Drew, Mgombane, Nyaruwa, & Foster, 1997; Rödlach, 2009). As in other countries, these are mostly volunteers who originally provided palliative care, but now support HIV testing, care and adherence (Schneider, Schaay, Dudley, Goliath, & Qukula, 2015).

The ZENITH randomised controlled trial tested CHW-delivered support for caregivers of recently diagnosed children aged 6–15 in Harare. The intervention consisted of 12–15 structured home visits during which CHWs counselled caregivers using a strengths-based case management approach (CDC, 2011) to address challenges to children’s care and treatment (Busza, Dauya, Bandason, Mujuru, & Ferrand, 2014). CHWs addressed caregivers’ treatment literacy, self-efficacy for children’s treatment, and willingness to disclose the child’s HIV status. CHWs also followed up children’s clinic appointments and linked families to welfare services, support groups for young people living with HIV, and programmes offering food aid or school fees.

Results of the ZENITH trial are reported elsewhere (Ferrand et al., 2017), but briefly, 166 children were randomly allocated to home visits and 168 to routine clinic care. At the end of follow up, 86% of enrolled children had initiated ART and a significantly higher proportion of children in the intervention arm were virologically suppressed 12 months after ART initiation. This was the first study to demonstrate improved virological outcomes among children following community-based support directed at caregivers.

## Methods

We conducted semi-structured interviews with caregivers of children receiving the intervention 12 and 18 months after enrolment. Caregivers were purposively selected for diversity in age, sex, relationship to the child, residence, and level of participation. At 12 months we interviewed 26 caregivers, but found that we reached thematic saturation after analysing 7–8 transcripts (i.e., we found broad agreement across themes with few outliers, suggesting additional sampling would be unlikely to yield new insights) (Bradley, Curry, & Devers, 2007; Hennink, Kaiser, & Marconi, 2017). We therefore reduced the number of caregivers interviewed at 18 months to 10. We interviewed different caregivers at each round. Topic guides explored caregivers’ decision to join the trial, experiences of home visits, and whether/how they felt the intervention influenced their care for a child living with HIV.

From caregivers interviewed at 12 months, we interviewed 15 children in their care. We recruited children aged 12 years and older who knew their status and had interacted with the visiting CHW. Children under 12 were not interviewed to comply with national ethics regulations. The topic guide asked children about their experience of learning their HIV status, living with HIV, willingness to talk to others about the challenges they face, and opinions of the intervention.

CHWs approached caregivers for interviews. Following their agreement, an independent female social scientist arranged interviews for a time and place convenient to respondents, usually their home. Interviews were conducted in the Shona language and lasted 30–60 min. Caregivers gave written consent for their interview and on behalf of any child in their care, with children giving verbal assent.

We interviewed all 20 CHWs who delivered the intervention at 12 and 18 months. The topic guide focused on the positive and negative aspects of providing home visits, and CHWs’ perspectives on barriers faced by caregivers and children in engaging with HIV services. The same female social scientist conducted all interviews, which she audio recorded, transcribed into Shona, and translated into English. Transcripts were entered into NVIVO 10 for thematic content analysis by the first author, who developed a “coding tree” for all references to HIV-related stigma. Findings were discussed during a 2-week analysis period by all authors, and cross-checked with the CHWs’ supervisor, leading to identification of high order codes on challenges posed by stigma to the intervention’s delivery and how these were managed.

Ethical approval was granted by the London School of Hygiene and Tropical Medicine, the Medical Research Council of Zimbabwe, and the Biomedical Research and Training Institute.

## Results

Findings are presented in three sections. First, we briefly characterise discrimination experienced by respondents and their perceptions of HIV stigma in the community to confirm its pervasive existence. Next, we focus on how fears of being stigmatised affected households’ acceptance of the ZENITH intervention. Third, we illustrate CHWs’ experiences of these challenges to delivery of activities and how they mitigated these.

### Anxiety around HIV discrimination

Both children and caregivers expressed anxiety about HIV in the family becoming known to others. These fears were grounded in experiences of gossip, isolation, and neglect related to HIV in their communities. Four children reported mistreatment, neglect, or derision by previous caregivers due to their HIV status, for example:

She [aunt] was now ill-treating me. … She was now shouting at me all the time. … She would ignore me when I greeted her. … She would treat me as if I was not her true relative. Even when washing clothes, she would bring out a big dish. We will be several girls, but she would just say “let her do it alone. Let her do everything” … and [to me] “You will not eat here!” [Girl, 16]

[My aunt] took care of me since I was in grade 4. … She viewed me as her son … But because I tested positive, the way she used to behave to me is now different. … Long ago we used to go to church together. We would do everything together. But now she can just say “you are not going to church. You are staying behind”. [Boy, 17]

All 15 interviewed children said fear of stigma discouraged them from disclosing their status to peers, either because they had been bullied and shunned or anticipated such discrimination:

Someone would come and throw sand at me. Another one would pour hot water at me and I would ignore them. … The neighbours go around gossiping, saying “the child from that place [household] is … HIV positive.” … You can actually hear them saying “ … do you know that children from there are HIV positive?” [Girl, 13]

I am worried that [my friend] will refuse to play with me ever again … because what friends do is that as soon as you tell them about your status … they might stigmatise you [Girl, 14]

Similarly, 11 caregivers described having witnessed or experienced HIV-related stigmatisation.

When this disease started we saw it. When a person left a plate of sadza it would be thrown away, even not given to the chickens [because]they would say “our chickens will die when people started falling sick”. … Even when someone left some rice, it would be thrown away. [Aunt of 14-year old girl, midline]

Caregivers living with HIV particularly felt that having suffered from discrimination themselves, they wanted to protect children.

[Community members] would laugh. They would laugh at me while walking along the road. … I stay with some people but during the first days when they knew about my status they didn’t even want to touch my cup. … so you feel that as a child, she might get affected. She might lose friends [Mother of 11-year old girl, midline]

### Effects of stigma on participation in the intervention

Concerns about HIV stigma had implications for the intervention’s feasibility. Five families refused CHW visits altogether, while others avoided letting CHWs enter their homes because they worried others would guess the purpose of the visits.

One [caregiver] is saying she will be at work and she cannot talk to me. She actually said “if you want to see me you will have to see me around 9, 10 in the evening.” That is when she will be back. It is not possible [for me] to leave home at night … it’s not feasible. … That is her way of refusing [visits] [CHW #9, female, midline]

We used to meet at the hall so that people at this house would not know about it. No one here knows except me, my one grandchild … [and] her uncle who is in South Africa [Grandmother of 11-year old girl, endline]

An explicit task of CHWs was to encourage caregivers and children to talk openly about HIV within family and beyond. CHWs conducted sessions on early, timely disclosure to children, benefits of finding supportive family/community members to assist with the child’s care, and other household members’ testing for HIV. These sessions proved challenging, as caregivers and children resisted key messages.

Yes, he [CHW] told us that we should tell friends, but … there are some that cannot be told. If you tell them it will spread it everywhere … it is better to keep quiet [Sister of 16-year old girl, midline]

Child: “No one knows. Therefore no one discriminates against me.”

Interviewer: “Why isn’t there someone else who is aware [of your status]?”

Child: “You will be stigmatized as if you are a disgusting person.” [Girl, 14]

CHWs feared that if children did not understand their condition, treatment adherence would suffer. CHWs felt caregivers hid the reason for ART to avoid mentioning HIV, while caregivers perceived this as a strategy to avoid discrimination.

They told her that the drugs are for asthma. You should just take them, but they are for asthma. The child will say “aunt, I am taking these tablets, but I do not know their purpose. Mama told me that they are for asthma, but I do not know their purpose”. [CHW # 6, Female, midline]

I just tell him “you are taking medication that is similar to mine for BP [high blood pressure], headaches, etc. It is normal”. I have never sat down with him and had a deep discussion. I am worried that … when he goes to school he will be telling other children at school that he is like this [HIV+]. [Aunt of 10-year old boy, endline]

### Responses by community health workers

CHWs adapted their activities to circumvent stigma-related barriers. First, they agreed to meet caregivers outside their homes, such as at a local market or community hall. This led to CHWs working in locations where they did not feel able to conduct activities as intended.

They do not want it to be known that they are HIV positive. This is a challenge that we have noticed. We are meeting in places that are not suitable, why? Because the majority of people are protecting themselves. [CHW #11, male, midline]

CHWs would also pretend to be a relative dropping by for a visit to avoid attracting attention.

When you arrive, you will say “I am your sister, I am looking for my sister”. … Then there are also situations whereby she is not free [to talk] at the place where she is staying. … It is not possible to go inside and have a discussion. … You can actually see that the situation is tense. You will then have [to arrange] another unscheduled visit [CHW #20, midline]

Over time, once greater trust was established with CHWs, some caregivers gradually changed their minds and became more receptive to home visits.

At first, I would meet the parents in the park. They would phone me, “we will meet in the park”. They did not want to be visited at home. I think they faced stigma and discrimination. … They later allowed me after 9 months or so. It was almost a year. [CHW #11, male, midline]

CHWs were sympathetic to concerns about stigma. They were recruited from the intervention area, which ensured their familiarity with the context, but also meant they shared local attitudes toward HIV and could be complicit in sustaining fears of discrimination. While CHWs promoted disclosure as per the ZENITH intervention manual, they expressed anxiety around doing so too early or if children were unable to assess who could be trusted with the information.

People are supposed to disclose but they are still in denial. According to our manual you are now at the disclosure stage, [but] … you can’t say people should disclose when you can actually see that they are still in a state of shock [CHW #11, male, midline]

She will not understand it. Disclosure has to be step-by-step until we can see that when I ask this child she can understand it. … When she goes out she will be saying “I have been told that I am HIV positive”. She will face discrimination. [CHW #5, female, endline]

All CHWs described trying to counteract stigma, building on their own experiences of having changed attitude. They tried to dispel myths about HIV transmission, which they considered the root cause of ongoing discrimination.

At first you might face stigma and discrimination from families, but this is because they do not have the knowledge. … Some will have the same beliefs that [we CHW] once had. [CHW #6, female, midline]

When I visited her for the first time I think this grandmother was affected because she was now saying, “I had removed her cup and put it on top of the refrigerator, and her plate, and spoon which she uses alone”. I said ‘no grandmother, all those things that you have put on top of the refrigerator … take them back and put them among your other plates. It doesn’t mean that when a child is HIV positive, the plates and the cups will also become positive. There is nothing like that.’ So, she understood. [CHW #13, female, endline]

Both caregivers and children credited CHWs with reducing stigma by being warm and open with people living with HIV, and teaching family members that children living with HIV were no different from others.

I think that she is someone who is loving, who is always visiting me to check up on me. … I can say when she comes she shows me that she is someone who is free, and she does not discriminate against us. [Girl, 13]

Long ago when we were not knowledgeable … even us adults, we would say the one who is HIV positive is dying. … But now through knowledge, sometimes you can actually hear her [child] explaining to someone that AIDS and HIV are not the same. A person who is HIV positive is not sick, you see. [Foster mother of 15-year old girl, endline]

## Discussion

We interrogated qualitative data collected during the ZENITH trial to assess how children’s, caregivers’ and CHWs’ experiences of stigma affected implementation of the intervention. Our findings confirm that increased “normalisation” of HIV has not reduced related stigma as much as hoped (Roura et al., 2009; Treves-Kagan et al., 2016). As in other studies (Machine et al., 2016), we found children and caregivers were afraid of discrimination, and wary that home visits might expose their HIV status. These fears were rational, as HIV-related discrimination featured prominently in respondents’ life accounts, and reflects Zimbabwe’s HIV Stigma Index in which 65.5% of people living with HIV reported having ever experienced HIV-related stigma or discrimination (ZNPP+, 2014).

Pervasive fear of stigma affected delivery of the ZENITH intervention, which relied on regular home visits, and promoted disclosing children’s HIV status to themselves, family and community members. Although few families refused home visits outright, many were anxious about visits, particularly early on. Our findings highlight how HIV stigma can hinder “getting a foot over the threshold” in community-based support programmes, which are increasingly recommended as a means to increase HIV diagnosis and treatment coverage (Alamo et al., 2012; Geldsetzer et al., 2017; Mavhu et al., 2017).

Children’s care is complicated by its mediation by caregivers, who may be influenced by their own experiences of HIV-related stigma (Murray et al., 2017). Wishing to avoid discrimination reduced some caregivers’ willingness to accept CHWs’ messages. These caregivers seemed most affected by discrimination and thus least likely to engage with services. CHWs found it particularly difficult to encourage disclosure of HIV status to children. Research from diverse settings highlights that one significant reason adults do not disclose to children is out of fear of resulting discrimination (Krauss et al., 2016; Vreeman et al., 2015). This suggests that households potentially most in need of support may be the most difficult to reach.

Masquillier et al. note that CHWs reflect values and attitudes of the communities where they work (Masquillier et al., 2016). This resonates with our finding that CHWs shared anxieties about stigma, although they overcame these. Indeed, a strength of CHWs is their familiarity with the local context (Kok et al., 2016), but this means they may themselves resist some intervention messages. We found, however, that CHWs adapted to challenges encountered by agreeing to meet caregivers on their own terms, and tackling stigma head-on, although this was not originally part of the intervention. CHWs made additional, unscheduled visits if caregivers seemed unwilling to participate when CHW arrived, or the visit was terminated early on arrival of others who did not know the child’s HIV status. While these strategies proved successful, they added to CHWs’ workload. Because our intervention was delivered in trial conditions, it was possible to provide CHW with intensive supervision and mentoring, critical to maintaining their motivation (Hermann et al., 2009; Jaskiewicz & Tulenko, 2012). Under normal programmatic conditions, this level of support could prove more difficult.

There are several implications of our study for future community-based programming. Newly established programmes should expect initial resistance due to HIV-related stigma, possibly from households at greatest need. It may be possible to conduct stigma-reducing campaigns prior to targeted interventions (Fakolade, Adebayo, Anyanti, & Ankomah, 2010; Khumalo-Sakutukwa et al., 2008; Maman et al., 2014; Tedrow et al., 2012). Peer-to-peer strategies could also be employed (Denison et al., 2012; Stangl et al., 2013), such as pairing household members welcoming of home visits with those more resistant.

Finally, CHWs may require preparation for addressing concerns about stigma and counteracting it. Programmes may need to incorporate additional discussion and self-reflection to help CHWs identify attitudes that hinder their ability to delivery key intervention messages.
